# DNA methylation pattern of the goat *PITX1* gene and its effects on milk performance

**DOI:** 10.5194/aab-62-59-2019

**Published:** 2019-02-20

**Authors:** Haiyu Zhao, Sihuan Zhang, Xianfeng Wu, Chuanying Pan, Xiangchen Li, Chuzhao Lei, Hong Chen, Xianyong Lan

**Affiliations:** 1 College of Animal Science and Technology, Northwest A&F University, Yangling, Shaanxi 712100, China; 2 Institute of Beijing Animal Science and Veterinary, Chinese Academy of Agricultural Science, Beijing 100194, China

## Abstract

Paired-like homeodomain transcription factor 1 (*PITX1*) is a pivotal gene in the hypothalamic–pituitary–adrenal axis, which is a well-known pathway affecting lactation performance. The aim of this study was to analyze the DNA methylation profile of the *PITX1* gene and its relevance to milk performance in Xinong Saanen dairy goats; thus, potential epigenetic markers of lactation performance were identified. A total of 267 goat blood samples were divided into “low” and “high” groups according to two milk traits: the average milk yield (AMY) and the average milk density (AMD). One CpG island in the 3${}^{\prime }$-flanking region of the *PITX1* gene was identified as being related to milk performance. Fisher's exact test demonstrated that the methylation rates of the overall CpG island and the 3rd and 12th CpG-dinucleotide loci in the blood were significantly associated with the AMY, and the overall methylation rate of the high AMY group was relative hypomethylation compared with the low AMY group. The overall methylation rates of this CpG island in mammary gland tissue from dry and lactation periods again exhibited a significant difference: the lactation period showed relative hypomethylation compared with the dry period. Bioinformatic transcription factor binding site prediction identified some lactation performance related transcription factors in this CpG island, such as CTCF, STAT, SMAD, CDEF, SP1, and KLFS. Briefly, overall methylation changes of the CpG island in the *PITX1* gene are relevant to lactation performance, which will be valuable for future studies and epigenetic marker-assisted selection (eMAS) in the breeding of goats with respect to lactation performance.

## 1 Introduction

Lactation performance is a complex quantitative trait affected by several crucial factors including genetic background, nutrition, and environment (Carcangiu et al., 2018). Over the past decade, the influences of genetic variations on milk performance in animals have been extensively studied (Koufariotis et al., 2018). Recently an increasing number of studies have demonstrated that nutritional or environmental changes contribute to changes in inheritable epigenetic modifications, such as DNA methylation (Lan et al., 2013a; Chavatte-Palmer et al., 2018; Kwan et al., 2018; Yuan et al., 2018). As one of the most important and common types of epigenetic modification, DNA methylation plays a significant role in regulating biological processes such as milk production and organ development (Hwang et al., 2017; Fleming et al., 2018; Jessop et al., 2018). In recent years, gene methylation involved in vital biological functions has been well-studied in animals (Ibeagha-Awemu and Zhao, 2015). However, to date, no association has been established between epigenetic modifications and lactation performance.

It is well-known that lactation performance is regulated by several critical pathways, such as the hypothalamic–pituitary–adrenal (HPA) axis pathway (*PITX2/PITX1–HESX1–LHX3/LHX4–PROP1–POU1F1*), which regulates the expression of *GH* (growth hormone) and *PRL* (prolactin) (Davis et al., 2010). As a member of the paired-like homeodomain transcription factor (PITX) family, the *PITX1* gene is located upstream of the HPA axis pathway and plays a critical role in pituitary organogenesis (Ma et al., 2017). During early pituitary development, *PITX1* activates the transcription of couples of pituitary hormone genes and transcription factor genes such as the LIM family, the POU family, and the SIX family (Poulin et al., 2000). Notably, *PITX1* has been characterized as an activator of the *POMC* gene and a partner of Pit1 causing differential expression of *GH*, *PRL*, and *TSH*, and thereby affecting milk performance in animals (Carvalho et al., 2006). In 2013, both the *PITX1* gene and another *PITX* family member, *PITX2*, were identified as being associated with milk performance in dairy goats (Lan et al., 2013b; Zhao et al., 2013).

Therefore, this study aimed at exploring the DNA methylation changes at the CpG islands within *PITX1* gene, as well as their relevance to milk performance. The present study is important and necessary for exploring the epigenetic regulation of lactation and developing potential epigenetic markers for improving milk performance through epigenetic marker-assisted selection (eMAS) in breeding.

## 2 Materials and methods

Experimental animals and the procedures performed in this study were approved by the Faculty Animal Policy and Welfare Committee of Northwest A & F University under contract (NWAFU-314020038). The care and use of experimental animals fully complied with local animal welfare laws, guidelines, and policies.

### 2.1 Animals, phenotypic data recording, and group classification

A total of 267 blood samples were obtained from healthy, unrelated adult female Xinong Saanen dairy goats, which were reared on the Chinese native dairy goat breeding farm in Qianyang County, Shaanxi, China (Zhao et al., 2013). The data regarding the average milk yield (AMY; kg) were obtained directly from the breeding farm, whereas the data regarding the average milk density (AMD) were measured by our study team using a MilkoScan FT120 (FOSS Corporation, Denmark) instrument (Lan et al., 2013b; Zhao et al., 2013). The relevant measurements of these two milk traits are listed in Table 1.

**Table 1:** Milk performance of the low and high groups of Xinong Saanen dairy goats.

Items	Average milk yield (kg)	Average milk density
Minimum values	272.4	1023
Maximum values	1057.6	1036.5
Data range	758.2	13.5
Mean	728.5	1029.2
SD	130.6	2.1
Kurtosis	0.021	1.397
Skewness	-0.346	0.005
Mean of low group	321.1${}^{B}$	1023.8${}^{B}$
SD of low group	68.8	1.1
Mean of high group	1024.8${}^{A}$	1035.5${}^{A}$
SD of high group	46.5	1.4
P values	P<0.001	P<0.001

The use of different letters (A, B) beside mean values in the same column signifies a significant difference at the P<0.001 level; SD stands for standard deviation.

Using the statistical probability (95.00 %) of the mean ±1.96 standard deviations for AMY and AMD, the low and high groups for each of the aforementioned milk traits were classified according to published literature (Pan et al., 2013). For the low or high group of each milk trait, two random pooled samples were constructed, and were named as follows: L-P1 (low-pool I), L-P2 (low-pool II), H-P1 (high-pool I), and H-P2 (high-pool II). Finally, eight DNA pools were constructed. It can be noted from Table 1 that the low group for each milk trait was significantly lower than average, whereas the high group was significantly higher than average. A t test was used to confirm the significance of the difference between the low and high groups, and the results validated that there were indeed significant differences between low and high groups for the AMY (kg) and the AMD (P<0.001; Table 1). In addition, five mammary gland tissue samples from Xinong Saanen dairy goats during a lactation period (n=2) and a dry period (n=3) were collected from another Xinong Saanen dairy goat farm, Fuping, Shaanxi Province, China (X. Zhang et al., 2017).

### 2.2 Genomic DNA extraction and bisulfite treatment

Genomic DNA samples from all chosen individuals were isolated as previously described (Cui et al., 2018; Wang et al., 2018). The DNA samples were then measured and diluted to a final concentration of 50 ng µL${}^{-1}$ (Wang et al., 2017; Q. Yang et al., 2017, 2018). Qualified DNA samples from the low and high groups were equally selected to construct genomic DNA pools. Each pool – containing 1000 ng mixed genomic DNA – was processed by sodium bisulfite using a QIAGEN EpiTect Fast DNA Bisulfite Kit (QIAGEN, Germany) and following the manufacture's instructions.

### 2.3 Bioinformatics analyses

The CpG islands within the goat *PITX1* gene were predicted using the MethPrimer website (http://www.urogene.org/methprimer/, last access: 10 January 2019) (Li and Dahiya, 2002), and the primers used to amplify the CpG islands were also designed using this website (Table 2).

**Table 2:** Primers used for PCR amplification.

Primers	Primer sequences (5${}^{\prime }$–3${}^{\prime }$)	Length (bp)	CpG numbers	Location (GeneID: 508754)
P1	F: GTTTAGATAGGAGTTGATTTTTGAG	126	8	5${}^{\prime }$-flanking region
	R: ATAACACACTAAATTCTCCCTC			
P2	F: GGGTGGTATTAATATAGGGTTTTAG	132	6	Intron 1
	R: CAATAATCACCTTCTAATCAAACTC			
P3	F: ATTAGTAGTTGGATTTGTGTAAGGG	106	10	Exon 3
	R: ACCCAATTATTATAAAAATACCCC			
P4	F: GTTATATGTTTTTGGGTGGGAT	403	24	3${}^{\prime }$-flanking region
	R: CTATTCAACCCAACTCCCTTAA			

To identify the possible trans-acting factors which might bind to specific CpG-dinucleotide locus, two respective authorized and reliable online bioinformatics softwares were used: TFSEARCH (Version 1.3) (http://mbs.cbrc.jp/research/db/TFSEARCH.html, last access: 19 June 2018) and the MatInspector database in Genomatix (http://www.genomatix.de, last access: 10 January 2019).

### 2.4 PCR amplification, cloning, and sequencing of the CpG island within the *PITX1* gene

The PCR reaction and the purification of the PCR products were performed as previously described (Pan et al., 2013). Purified fragments were cloned into the pGEM-T Easy Vector (Promega, WI, USA) system. The colony PCR was used to identify the positive inserts, which were sequenced via sequencing service (GenScript, Nanjing, China). The number of positive inserts for each pool sent for sequencing was around 10, according to the efficiency of ligation and transformation. The methylation status for the CpG island and each CpG-dinucleotide locus was measured by sequencing, and alignment analyses were carried out using DNAMAN software (version 7.0, Lynnon Biosoft, Vaudreuil, Quebec, Canada) (Lan et al., 2013a).

### 2.5 Statistical analysis

Comparisons of the methylation difference at each CpG-dinucleotide locus, the entire CpG island between the low and high groups for each milk trait, and the methylation status of mammary gland tissue from dry and lactation periods were analyzed using the Fisher's exact test ($\chi^{2}$-test) as previously described (Lan et al., 2013a).

## 3 Results

### 3.1 DNA methylation profile of the *PITX1* gene

**Figure 1:**
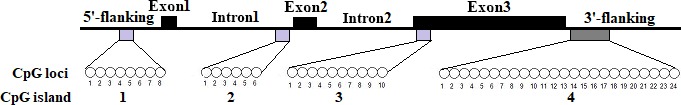
Gene structure and CpG islands distribution within the goat *PITX1* gene.

**Figure 2:**
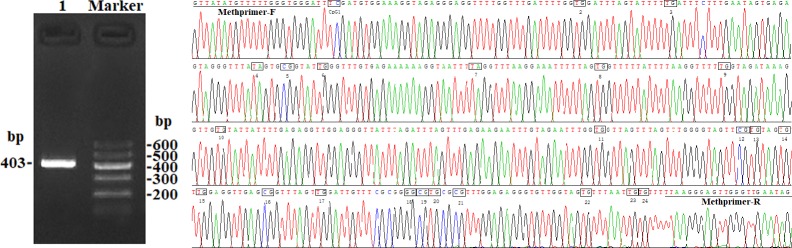
PCR electrophoresis diagram and bisulfite sequencing maps of the goat *PITX1* CpG island.

In this study, four CpG islands located at the 5${}^{\prime }$-flanking region, intron 1, exon 3, and 3${}^{\prime }$-flanking region of the *PITX1* gene were predicted (Fig. 1). A total of four pairs of methylation primers were designed to amplify these four CpG islands. Due to the complexity and low G and C bases content of bisulfite-processed genomic DNA as well as the low amplification efficiency for bisulfite PCR, only one 403 bp-CpG island (24 CpG-dinucleotide loci) located at the 3${}^{\prime }$-flanking region was accessible using the P4 primer (Fig. 2). At the available CpG island within the *PITX1* gene, about 10 positive inserts for each sample were sequenced. The alignments of the sequences demonstrated different overall methylation patterns in the low and high pools for the two milk traits; the overall methylation rate of these blood DNA pools varied from 22.46 % (high pool group for AMY) to 33.33 % (high pool group for the AMD) (Table 3). In addition, different overall methylation patterns were detected in mammary gland tissue from dry (57.29 %) and lactation (45.03 %) periods (Table 4).

**Table 3:** Methylation percentage and P value difference of each CpG-dinucleotide locus between the low and high groups.

Milk traits	Average milk yield			Average milk density		
CpG no.	Low group	High group	P value	Low group	High group	P value
CpG1	43.75 %	17.39 %	0.146	22.22 %	38.10 %	0.338
CpG2	56.25 %	34.78 %	0.209	66.67 %	52.38 %	0.380
CpG3	50.00 %	17.39 %	0.041	18.52 %	28.57 %	0.498
CpG4	18.75 %	8.70 %	0.631	0.00 %	0.00 %	1.000
CpG5	75.00 %	43.48 %	0.099	55.56 %	66.67 %	0.555
CpG6	0.00 %	0.00 %	1.000	0.00 %	0.00 %	1.000
CpG7	6.25 %	0.00 %	0.410	3.70 %	4.76 %	1.000
CpG8	6.25 %	8.70 %	1.000	25.93 %	28.57 %	1.000
CpG9	25.00 %	4.35 %	0.139	18.52 %	23.81 %	0.729
CpG10	43.75 %	30.43 %	0.503	44.44 %	42.86 %	1.000
CpG11	25.00 %	47.83 %	0.192	29.63 %	38.10 %	0.555
CpG12	0.00 %	47.83 %	0.001	37.04 %	19.05 %	0.214
CpG13	25.00 %	17.39 %	0.694	37.04 %	38.10 %	1.000
CpG14	25.00 %	30.43 %	1.000	0.00 %	4.76 %	0.438
CpG15	18.75 %	47.83 %	0.093	44.44 %	33.33 %	0.555
CpG16	43.75 %	17.39 %	0.146	44.44 %	42.86 %	1.000
CpG17	18.75 %	13.04 %	0.674	37.04 %	52.38 %	0.382
CpG18	12.50 %	13.04 %	1.000	22.22 %	23.81 %	1.000
CpG19	50.00 %	21.74 %	0.090	48.15 %	52.38 %	1.000
CpG20	37.50 %	17.39 %	0.264	59.26 %	42.86 %	0.383
CpG21	43.75 %	34.78 %	0.740	51.85 %	38.10 %	0.393
CpG22	43.75 %	21.74 %	0.174	44.44 %	57.14 %	0.561
CpG23	25.00 %	13.04 %	0.415	44.44 %	33.33 %	0.555
CpG24	6.25 %	30.43 %	0.109	29.63 %	38.10 %	0.555
Total	29.17 %	22.46 %	0.022	32.72 %	33.33 %	0.850

**Table 4:** Methylation percentage and P value difference for each CpG-dinucleotide locus between the mammary gland tissue from the dry and lactation periods.

Periods/CpG no.	Lactation	Dry	P value	Periods/CpG no.	Lactation	Dry	P value
CpG1	42.31 %	50.00 %	0.766	CpG13	38.46 %	55.00 %	0.372
CpG2	57.69 %	65.00 %	0.763	CpG14	30.77 %	55.00 %	0.135
CpG3	38.46 %	65.00 %	0.001	CpG15	42.31 %	55.00 %	0.552
CpG4	42.31 %	50.00 %	0.008	CpG16	34.62 %	60.00 %	0.136
CpG5	57.69 %	60.00 %	1.000	CpG17	38.46 %	50.00 %	0.552
CpG6	42.31 %	50.00 %	0.766	CpG18	19.23 %	30.00 %	0.494
CpG7	42.31 %	40.00 %	1.000	CpG19	42.31 %	55.00 %	0.552
CpG8	34.62 %	55.00 %	0.233	CpG20	57.69 %	70.00 %	0.540
CpG9	50.00 %	55.00 %	0.774	CpG21	53.85 %	65.00 %	0.551
CpG10	53.85 %	65.00 %	0.551	CpG22	46.15 %	70.00 %	0.139
CpG11	65.38 %	70.00 %	1.000	CpG23	46.15 %	65.00 %	0.244
CpG12	53.85 %	60.00 %	0.769	CpG24	50.00 %	60.00 %	0.561
				Total	45.03 %	57.29 %	$6.10\times 10^{-5}$

### 3.2 DNA methylation differences between the low and high groups for the two milk traits

**Figure 3:**
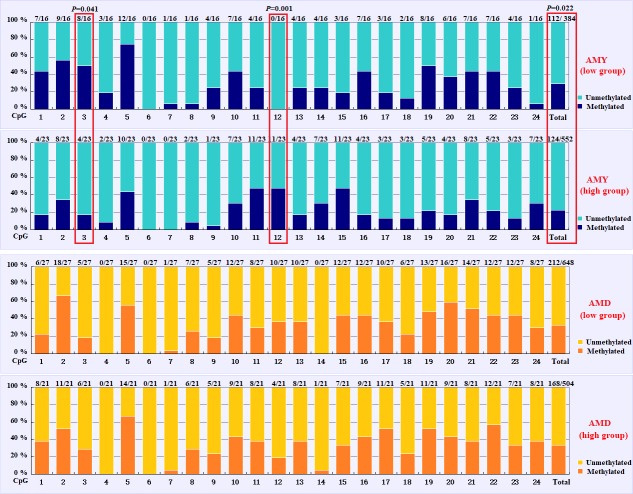
Comparisons of the DNA methylation differences for each CpG locus between the low and high groups for the two lactation traits. The numbers (X/Y) above each bar represent the ratio of methylation versus total colonies, and the red frames represent significant differences. AMY refers to the average milk yield, and AMD refers to the average milk density.

Based on the methylation status of each CpG-dinucleotide locus, methylation differences between the low and high groups were evaluated according to Fisher's exact test ($\chi^{2}$-test). As seen from Table 3 and Fig. 3, the overall methylation level of the CpG island within the *PITX1* gene was significantly associated with the AMY (P=0.022). Specifically, the overall methylated percentage of the high AMY group (22.46 %) was significantly lower than that of the low group (29.17 %). For individual CpG-dinucleotide locus, the methylation status of the 3rd CpG-dinucleotide locus was significantly associated with the AMY (P=0.041), and the methylation percentage of this locus from high group (17.39 %) was significantly lower than that from low group (50.00 %); on the contrary, the methylation status of the 12th CpG-dinucleotide locus showed a negative association with the AMY, and the methylation rate of the high group (47.83 %) was significantly higher than that of the low group (0 % (Table 3 and Fig. 3). However, no significant relevance was found between methylation changes and the AMD.

### 3.3 DNA methylation differences between mammary gland tissue from dry and lactation periods

**Figure 4:**
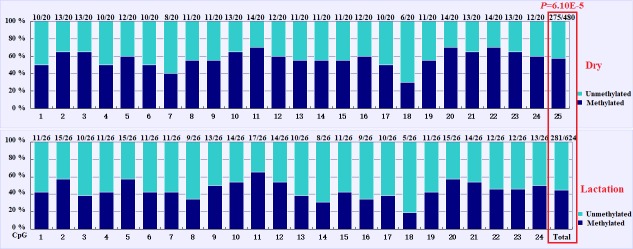
Comparisons of the DNA methylation differences for each CpG locus for mammary gland tissue from dry and lactation periods. The numbers (X/Y) above each bar represent the ratio of methylation versus total colonies, and the red frame represents a significant difference.

The overall DNA methylation rates of the CpG island in mammary gland tissue from dry and lactation periods exhibited a significant difference ($P=6.10\times 10^{-5}$); mammary gland tissue from the dry period showed hypermethylation (57.29 %), whereas mammary gland tissue from the lactation period showed relative hypomethylation (45.03 %) (Table 4 and Fig. 4). However, no significant relevance was identified on each CpG-dinucleotide locus in mammary gland tissue from the dry and lactation periods.

### 3.4 Possible trans-acting factors binding to the CpG-dinucleotide loci

**Figure 5:**
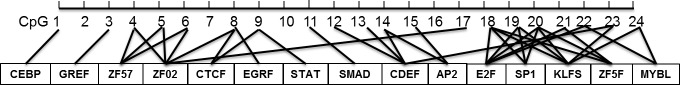
Prediction of the possible binding transcription factors for each CpG-dinucleotide locus in the CpG island within the goat *PITX1* gene.

According to the bioinformatical analyses, several crucial transcription factors, such as CTCF, STAT, SMAD, CDEF, SP1, KLFS, and zinc finger transcription factors were predicted to bind to the CpG-dinucleotide loci of this CpG island within *PITX1* (Fig. 5). In this CpG island, 24 CpG-dinucleotide loci were sparsely localized in the 403 bp region, and potential transcription factors were predicted to bind to the nearby area of almost every CpG-dinucleotide loci.

## 4 Discussion

Extensive DNA methylation studies have focused on the CpG islands located at the promoter region, as the methylation of promoter CpG islands could result in self-perpetuating gene silencing (Moore et al., 2013). However, an increasing number of studies have found that a far greater proportion of methylation occurs across the whole gene (Jones, 2012). Genome-wide studies have revealed that gene body methylation is evolutionarily conserved (Keller et al., 2016). Therefore, this work attempted to study the methylation of four possible CpG islands across the whole *PITX1* gene in a group Xinong Saanen dairy goats. However, only the CpG island located at the 3${}^{\prime }$-flanking region was available. It is well known that 3${}^{\prime }$-flanking regions are also important in gene regulation (Yang et al., 2016).

Recently, numerous epigenetic markers have been identified as being involved in biological functions and livestock traits using whole genome bisulfite sequencing (WGBS) and bisulfite sequencing methods (Y. Yang et al., 2017; Sarova et al., 2018). Fang et al. (2017) uncovered 331 meat quality traits related to candidate differentially methylated regions (DMRs) in Japanese Black (Wagyu) and Chinese Red Steppes cattle. Y. Zhang et al. (2017) revealed 10 DMR-related genes (DMGs) by analyzing the sequencing results of Hu sheep ovaries, which were likely related to the prolificacy of Hu sheep. Li et al. (2018) identified potential DMRs and DMGs that were involved in hair follicle development and growth in cashmere goats. Dechow and Liu (2018) detected the DNA methylation patterns in peripheral blood mononuclear cells of Holstein cattle with variable milk yield, and revealed some potential epigenetic variation related to milk performance. With the rapid development of science, more and more crucial genetic and epigenetic markers will be discovered, which will provide abundant information for animal breeding and biological research.

Numerous genes have been proved to be regulated by DNA methylation, and there are a few possible mechanisms by which this can occur (Mattern et al., 2017). The methyl-group in CpG islands might result in compact and inactive chromatin structure, recruiting methyl-CpG binding proteins or preventing relevant transcription factors from binding, which inhibits the initiation or proceeding of transcription (Kang et al., 2015). In this study, we designed three pairs of primers to detect the expression of goat *PITX1*. However, we failed to detect the expression of goat *PITX1* in mammary gland tissue and other tissues (heart, liver, spleen, lung, kidney, muscle, and adipose tissue – data not shown). Furthermore, according to the Ensembl, NCBI, and BioGPS databases, there is little expression of *PITX1* in the adult mammary glands of humans, mice, and sheep. Even though there was no expression of *PITX1* in mammary gland tissue, we speculate that the epigenetic modification of *PITX1* can still be an useful epigenetic marker for improving lactation performance in the process of goat epigenetic marker-assisted selection in breeding at the DNA level.

In addition, these findings have also been corroborated by the biological functions of some possible transcription factors predicted in this study, including the following: (1) CTCF can regulate gene expression by binding to the gene imprinting control region (ICR) (Dunn and Davie, 2003); (2) the STAT family of proteins are the main components in the JAK–STAT pathway, which is critical to the proper development and function of the mammary epithelial tissue (Haricharan and Li, 2014); (3) SMAD proteins are Smad signaling components – in mammary epithelial cells, Smad signaling acts to antagonize JAK–STAT signaling to regulate mammary gland differentiation (Cocolakis et al., 2008); (4) CDEF could regulate the cell cycle of epithelial cell (Müller and Engeland, 2010); and (5) SP1 and KLFS could regulate the expression of genes containing GC-rich promoters (Kaczynski et al., 2003), and these transcription factors could influence the function of *PITX1* and therefore affect lactation performance.

## 5 Conclusion

In this study, a functional CpG island at the 3${}^{\prime }$ flanking region of goat *PITX1* gene was identified. In this region, the overall methylation rate of the high AMY group showed relative hypomethylation compared with the low AMY yield group; the overall methylation rate of mammary gland tissue from the lactation period showed hypomethylation compared with the dry period. These results will provide epigenetic markers for improving lactation performance with regards to the process of goat epigenetic marker-assisted selection in breeding.

## Data Availability

The original data are available upon request to the corresponding author.

## Appendix A Abbreviations

**Table:**

PITX	Paired-like homeodomain transcription factor
*PITX1*	Paired-like homeodomain transcription factor 1
AR	Androgen receptor
eMAS	Epigenetic marker-assisted selection
HPA axis	Hypothalamic–pituitary–adrenal axis
SD	Standard deviation
AMY	Average milk yield
AMD	Average milk density
WGBS	Whole genome bisulfite sequencing
DMRs	Differentially methylated regions
DMGs	Differentially methylated region-related genes
